# The development of a mechanical device to stretch skeletal muscle of young and old rats

**DOI:** 10.6061/clinics/2019/e629

**Published:** 2019-09-04

**Authors:** Talita Gianello Gnoato Zotz, Rafael Zotz, Ana Tereza Bittencourt Guimarães, Eduard Goossen, Anna Raquel Silveira Gomes

**Affiliations:** I Departamento de Prevencao e Reabilitacao em Fisioterapia, Universidade Federal do Parana, Curitiba, PR, BR; II Bioterio Central, Pontificia Universidade Católica do Parana, Curitiba, PR, BR; III Universidade Estadual do Oeste do Parana, Cascavel, PR, BR; IV Departamento de Prevencao e Reabilitacao em Fisioterapia, Programa de Mestrado e Doutorado em Educacao Fisica, Universidade Federal do Parana, Curitiba, PR, BR

**Keywords:** Muscle Stretching Exercise, Rats, Force, Aging

## Abstract

**OBJECTIVE:**

How much force is needed to stretch skeletal muscle is still unknown. The aim of this study was to develop a device that mechanically stretches rat muscle to compare the force (N) required to stretch the soleus muscle of young and aged rats and the tibio-tarsal angle joint at neutral and stretched positions.

**METHODS:**

Twelve female Wistar rats were divided into two groups: a young group (YG, n=6, 311±11 g) of rats 3 months old and an aged group (AG, n=6, 351±43 g) of rats 15 months old. The left soleus muscle was mechanically held in full dorsal flexion and submitted to mechanical passive stretching: 1 bout of 10 repetitions, each repetition lasted 60 seconds with an interval of 45 seconds between repetitions, performed once a day, twice a week, for 1 week. The force required during stretching was measured by a load cell, and the tibio-tarsal angle joint was measured by photometry.

**RESULTS:**

The load cell calibration showed excellent reliability, as confirmed by the intraclass correlation coefficient value of 0.93. A decrease in delta force was found in the comparison between YG and AG (0.11±0.03 N *vs* 0.08±0.02 N, *p*<0.05, repeated measures ANOVA). There was no difference between the YG and the AG in the tibio-tarsal angle at resting position (87.1±3.8° *vs* 87.1±3.5°, *p*=0.35, Kruskal Wallis) and at the end of the stretching protocol (43.9±4.4° *vs* 42.6±3.4°, *p*=0.57, Kruskal Wallis).

**CONCLUSION:**

The device presented in this study is able to monitor the force necessary to stretch hindlimb rat muscles. Aged rats required less force than young rats to stretch the soleus muscle, and there was no difference regarding the tibio-tarsal angle between the two groups.

## INTRODUCTION

Muscle stretching is defined as an exercise that involves the application of a force to improve the resistance of connective tissue by increasing the length of a muscle-tendon unit and the range of motion (ROM) 1. This type of exercise promotes physical benefits in young and old people, including enhanced flexibility and/or ROM 2,3, fascicle length 4, functional capacity 5, and performance 6.

Animal studies show that manual passive stretching is sufficient to maintain and/or increase joint ROM by promoting increased serial sarcomere number and inhibiting connective tissue proliferation and atrophy 7,8. Nevertheless, in animal studies, the tension to stretch the soleus muscle is applied by a manual force that is sufficient to hold the maximum limit of joints' ROM; the amount of force to promote muscle stretching, however, has not been monitored 9.

Instruments have been designed to monitor skeletal muscle stretching in animals 10-13. For instance, to stretch the extensor digitorum longus and other foot extensor muscles, an apparatus was developed to provide a constant moment-arm to apply torque to the ankle joint and allow the extensor muscles that move the foot to be stretched under a constant force. However, the apparatus targeted only young mice (not rats), focusing on the stretching of foot extensors (not ankle flexor muscles); it also did not contain adjustable parts to allow stretching on animals of different ages, sizes and weights 10.

Another study created an apparatus aiming to stretch the rat soleus, an ankle flexor muscle. The young rat hindlimb was stabilized by fixing its foot onto a platform that was connected to a movable wire. The stretching amplitude and frequency were controlled by the stepping motor. Although the apparatus was able to control these ranges through a goniometer, the force applied to stretch the soleus muscle had not been measured 13.

In other studies, another device was used to stretch the rat soleus muscle. The young rat foot was held in dorsiflexion by a spring balancer set at a force of 0.3 N. However, the hip and knee joint positions were not stabilized, and the force applied during the stretching was equal for all rats 11,12.

Due to the reasons mentioned above, the authors suggest that there is a lack of research aiming to measure the amount of force applied to stretch rat calf muscles, which justifies the development of an apparatus for this goal. Additionally, the intensity of stretching is relatively underresearched, and its effects on musculo-tendinous tissue are largely unknown 14. Animal studies allow the investigation of the histomorphometry and cellular mechanisms involved in skeletal muscle adaptation, which is limited in human studies, thus enhancing the importance of a piece of equipment to monitor the stretching force. In this sense, it is still unknown how much force should be applied to stretch the skeletal muscle of young and aged rats. Thus, the objective of this study was (1) to develop a device to mechanically stretch rat skeletal muscle; (2) to compare the force necessary to stretch soleus muscle of young and aged rats; and (3) to compare the tibio-tarsal angle joint in neutral and stretched positions.

## MATERIALS AND METHODS

This study was divided into three phases: 1) Device development; 2) load cell calibration; 3) stretching protocol application on young and aged female rats.

### Experimental Design

The device was tested to mechanically stretch the soleus muscle of young and aged rats. Thus, twelve female Wistar rats (*Rattus norvegicus albinus*) were used and divided into two groups: a young group (YG, n=6) of 3-month-old animals and an aged group (AG, n=6) of 15-month-old animals 15-17. The project followed the international ethics standard for animal experiments 18 and was approved by the Ethics Committee on Animal Use of the Pontifical Catholic University of Paraná (PUCPR) (PROTOCOL n° 732/2012). To determine the sample size, the minimum sample number of 6 individuals per experimental group was followed because a homogeneous population 19 of laboratory animals was considered. In this arrangement, the probability of having each rat present a distinct category would be 16%.

The female rats were kept inside a bioterium in standard plastic cages under controlled environmental conditions (luminosity: bright/dark 12/hour cycle) with free access to food pellets and water.

### Device Development

The device presented in this study was developed to measure the force applied when stretching exercises are carried out. For this reason, it included a load cell that is able to register the necessary force (in grams) to stretch the soleus muscle.

**Figure 1:**
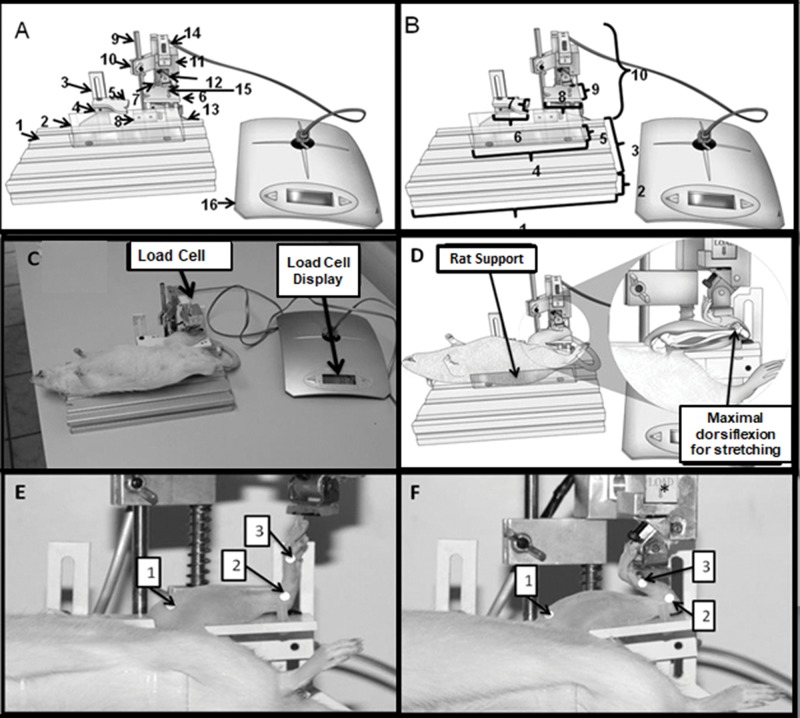
**A.** Device to stretch the soleus muscle of rats. 1- Base made of aluminum material; 2- part used to support the animal's body; 3- bar with an adjustable horizontal and vertical support guide; 4-part for adjusting the position of the hip joint; 5- base for supporting the thigh; 6- base for sustaining the shank; 7- part that vertically moves the load cell system; 8- support for the load cell; 9- axis for adjusting the shank position; 10- part for vertical manipulation of the load cell system; 11- load cell in the pendulum. 12- belt for fixing the tibio-tarsal joint; 13- load cell display. **B**. Device dimensions. 1- 23.4 cm; 2- 3 cm; 3- 12 cm; 4- 13.5 cm; 5- 3.5 cm; 6- 3.3 cm; 7- 3.3 cm; 8- 4.2 cm; 9- 4 cm; 10- 13.0 cm. **C.** Image showing an animal on the device with the left hindlimb positioned for soleus muscle stretching; **D**. Schematic drawing of the details of the rat support and maximal dorsiflexion during stretching. **E**. Knee and tibio-tarsal joints positioned at 90° and markers: 1- tibia (medial condyle); 2- medial malleolus; 3- head of the first metatarsal bone; **F**. Tibio-tarsal joint at maximum dorsiflexion during the stretching protocol and markers: 1- tibia (medial condyle); 2- medial malleolus; 3- head of the first metatarsal bone.

To situate the animal in the proper position so that its muscle could be stretched, the device was manufactured with adjustable parts. Its adaptable portions allow the rat to fit into the device independent of its size and weight and to have its joints adjusted according to the target muscle being stretched. Figure 1 demonstrates the stretching device and depicts its adjustable parts.

The device containing the load cell for measuring the force applied to induce soleus muscle stretching in rats is also demonstrated in Figure 1. The parts and dimensions of the device are labeled in the legend of Figures 1A and 1B. Finally, the device is registered under patent (number BR1020150205740).

### Load Cell Calibration

The load cell was used to measure the force applied to stretch the muscle. The load cell calibration was verified according to Doebelin 20. Regardless of how good the characteristics of a measuring system are, they can always display errors caused not only by internal factors but also by external factors. Properly characterizing uncertainties associated with these errors is important so the measurement results can be securely estimated. Because the errors of a measurement system can be analyzed or numerically estimated in some cases, experimental procedures were used to determine the reliability of its readings 20. This process is known as calibration, during which the values measured by the device were correlated with the magnitude being calculated.

**Figure 2:**
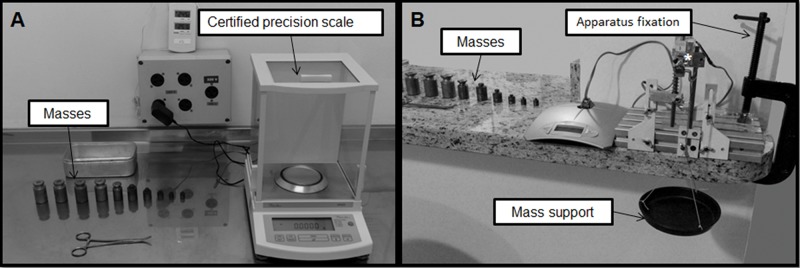
Sequence to load cell calibration of the stretching device. **A.** Calibration of masses with a precision scale. **B.** load cell calibration of the device. *Load cell used to measure the force applied to promote the stretching of the soleus muscle.

In the case of the stretching device, the load cell reads in grams (g) with the maximum value able to be read being 1.09 g. The device was calibrated using the masses that had already been weighted. First, these masses were weighted seven times by a certified precision scale (Figure 2A); these values were used to calculate the average of the masses to check the reliability of the device's load cell 21. Then, due to the pendulum position of the load cell, a container was hung to support the masses (Figure 2B). After that, the device was set on the countertop to avoid vibration during the measurement.

Subsequently, each mass was placed into the support of the precision scale to check the load cell until all the masses were included. Afterward, each mass was placed, one by one – from the last to the first – and the load cell reading was verified. This procedure was repeated seven times, and the average of the values was calculated to estimate the reliability of the cell reading 20. Linearity was checked as part of the procedures to investigate the reliability of the load cell calibration.

### Hind Limb Photometry

Photometry was used to evaluate the angle formed at the tibio-tarsal joint before and at the end of stretching protocol. To do this, the animal was placed on the device, which was set on a countertop 1.06 meters above the floor. A digital camera was used (Canon EOS rebel 600 d, Canon brand lens and macro lens EF 100 mm 1: 2.8 L IS USM); the camera was positioned on a tripod 1.07 meters high, perpendicular to the sagittal plane of the animal at a focal length of 80 cm from the device with the focus aimed at the tibia 22,23. Photographs were captured with a flash and 18 megapixel resolution.

To ensure that the tibio-tarsal joint was kept in contact with the device support surface, a belt to keep the ankle fixed was wrapped around that joint to keep it stable (Figure 1E). The markers used were reflective stickers with a diameter of 4 mm. The animals had been previously trichotomized to facilitate sticker adherence to the skin.

A set of three stickers were stuck at the following anatomical points: tibia (medial condyle), medial malleolus, and head of the first metatarsal bone. The angle formed by straight segments, traced from the tibia and metatarsal segments, was calculated from the coordinate system obtained by Image J program 22. Three photographs were taken at the resting position (Figure 1E) and stretching (Figure 1F) position for each animal. Therefore, six images were collected per animal, resulting in 72 images for the analysis. The mean value of the three measurements was considered during the analysis (i.e., one measurement of each photograph was taken at each position (three photographs at resting position and three at stretching position) per animal).

### Stretching Protocol

To perform the muscle stretching exercise, the animal was first weighed (on a Mettler/Toledo scale with a capacity of 25 g to 3 kg) and then anesthetized with an intraperitoneal injection of 80 mg/kg ketamine and 8 mg/kg xylazine. Next, the animal was positioned on the stretching device with the tibio-tarsal joint at its maximum dorsal flexion to stretch the soleus muscle 24,25. The stretching protocol consisted of a bout of 10 repetitions 25; each repetition lasted 1 minute, with 45 second intervals between each repetition, and the stretching protocol was controlled by a chronometer (Technos) 25,26. The repetitions were carried out once a day, twice a week (on Monday and Thursday) – maintaining an interval of two days between each stretching session – over the course of one week. All animals of both groups underwent the stretching protocol.

The rat was positioned in a supine position, the ankle joint was placed on the base to sustain the shank, and a compressive force was applied to the plantar region of the paw 24 (Figure 1C and 1D).

The stretching protocol was conducted in the following sequence: 1) the device was installed; 2) the rat was positioned in the device with its knee and tibio-tarsal joints fixed at 90° on the device's support; 3) the tibio-tarsal joint was positioned in a full dorsiflexion to promote soleus muscle stretching (Figure 1D) for one minute; the force was monitored by the load cell display (Figure 1A). After each stretching repetition, the tibio-tarsal joint returned to the neutral position to rest 24.

The data resulting from the reading of the load cell were recorded at the beginning and end of each stretching repetition to determine the force required to stretch soleus muscle in both groups (YG and AG).

### Statistical Analysis

The intraclass correlation coefficient (ICC) was calculated using SPSS, version 20. The standard error of measurement (SEM) was calculated using the following equation: standard deviation multiplied by √(1-ICC).

The data related to body weight, load cell calibration, and force were analyzed. The normality of the data was determined using the Shapiro-Wilk test, and the homogeneity was determined by the Levene test. Repeated measures ANOVA was used to compare the initial and final body weights between groups. The output and the weight of the load cell were transformed using a logarithmic function of log (x+1) and evaluated with Pearson's correlation and linear regression. Repeated measures ANOVA was used to compare the delta force (final minus initial force), followed by the Tukey-HSD test for intergroups (young and aged groups) and intragroup comparisons (repetitions). The level of significance was set to 5% for all comparisons; these analyses were performed using R software.

## RESULTS

### Body Weight

An increase in the final body weight was found compared with the initial values in the YG (315.3±9.2 g *vs* 311±11.3 g, *p*=0.02, Tukey's test), but no increase was found in the AG (352.3±42.4 g *vs* 351±43.2 g, *p*=0.36, *p*=0.83, Tukeýs test) or between the YG and the AG (*p*=0.49, Tukey's test).

### Load Cell Calibration

**Figure 3:**
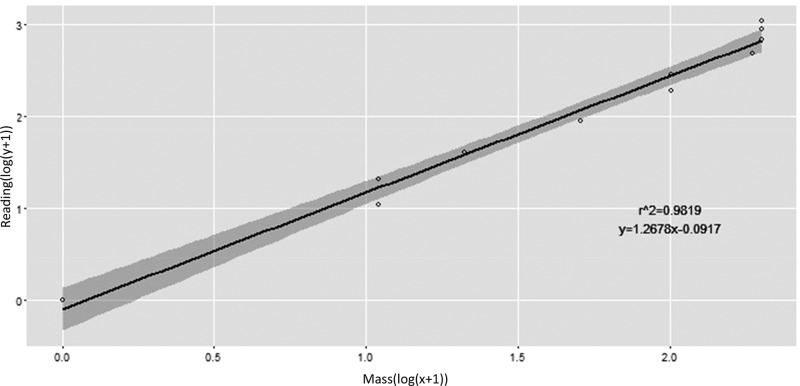
Linear trend of load cell calibration.

The load cell calibration showed excellent reliability, as confirmed by the ICC value of 0.93 with an SEM of 0.003. Regarding the regression line, 98.19% of the variation in the output data load cell might be explained by the mass load cell (y=1.2678*x-0.0917; r^2^=0.9819, t=22.12; *p*<0.01; Figure 3).

### Force Behavior to Stretch

There was no difference between the first and the last (tenth) values registered by the load cell related to the force applied to stretch the soleus muscle of the YG or the AG (Table 1).

No significant intragroup difference was found when the delta force was compared (F_9,90_=0.95; *p*=0.48). Moreover, the delta force was similar between the YG and the AG in each repetition, but when the delta force of all repetitions was compared, the YG had a higher mean than the AG (0.11±0.03 N *vs* 0.08±0.02 N, *p*=0.00) (Table 2).

### Tibio-Tarsal Joint Angle

There was no difference between the YG and the AG in the tibio-tarsal angle in the resting position (87.1±3.8° *vs* 87.1±3.5°, *p*=0.35, Kruskal Wallis) or at the end of the stretching protocol (43.9±4.4° *vs* 42.6±3.4°, *p*= 0.57, Kruskal Wallis test).

## DISCUSSION

The main objective of this study was to develop a device to monitor the force applied during muscle stretching exercise in rats. The outcomes of this study show that the developed device efficiently could execute stretching exercises mechanically, allowing the amount of force to be controlled during the repetitions in both young and aged rats. Furthermore, the reliability and linearity of the load cell during the stretching exercises were verified. Additionally, less force was necessary to stretch the soleus of aged rats compared with young rats.

Manual stretching has been used in most studies on this topic. In previous studies, it was not possible to quantify the force applied when inducing muscle lengthening in rats 7,8,25-29. Thus, the device described in this paper achieved its initial purpose of allowing researchers to control the force applied during skeletal muscle stretching.

Other authors developed a dynamometer that allowed them to quantify the static and dynamic plantar flexor's muscle response in anaesthetized rats *in vivo* by automation of the testing and data-acquisition procedures. The dynamometer could operate in isometric, isovelocity, or controlled non-isokinetic torque. Moreover, the ROM of the ankle joint and electrical stimulation of the rat muscles were controlled individually and independently. As the dynamometer was specifically designed to control velocity, angle and torque, it did not indicate the force needed to stretch the muscle 21. For this reason, the device developed in the present study was designed to monitor the force used to stretch the soleus muscle of young and aged rats.

Pratt and Lovering 30 described an *in vivo* animal model of the quadriceps for measuring torque that could produce a reliable muscle injury and then follow-up muscle recovery of the same animal over time. The authors also described a second model used for the direct measurement of force from an isolated quadriceps muscle *in situ*. Hence, the device presented in the current paper was designed to stretch the muscle group of the ankle in both young and aged rats.

Black et al. 10 performed stretching of the extensor digitorum longus (EDL) muscle of mice using a specifically designed piece of equipment. The rationale behind their apparatus design was that the rotary platform would provide a constant moment-arm to apply torque to the ankle joint and allow the muscles that move the foot to be stretched by a constant force. The foot platform was rotated by slowly moving the force transducer backwards (away from the mouse) to stretch the lower hindlimb extensor muscles, including the EDL. The device developed in the present study differs from the equipment built by Black et al. 10 in several aspects as it contains adjustable parts for different rat sizes and weights. This feature enables the assessor to individually monitor the force applied to stretch the soleus muscle. That is, the force applied to promote muscle stretching is not predetermined; it is established for each rat, and it can be checked at each repetition, allowing assessors to verify the possible force changes during stretching. In this sense, the device presented in this paper goes further, as it also allows researchers to stretch the gastrocnemius, since the parts are adaptable to the hindlimb joints.

Inoue et al. 13 used custom-built stretching equipment to perform a cyclically stretching exercise on rat soleus muscles. The device was able to monitor the amplitude and frequency of stretching, but the hip and knee joint were not fixed to isolate soleus muscle lengthening. Alternatively, the device developed in the present study contains parts to secure hip, knee and tibio-tarsal joints in position, which ensures isolated stretching of the soleus muscle. In addition, the rat is able to remain at the supine position on the device to mimic the muscle stretching exercise position carried out on humans.

Other authors performed daily, prolonged passive soleus muscle stretching using a spring balancer set at a force of 30 g 11,12. However, the hip and knee were not fixed during stretching, which impaired the isolation of the soleus muscle. The device developed in the present study measures the necessary force to stretch each animal during each repetition (i.e., individually, without predetermined force), as devices in other studies have required.

In the current study, the force applied to promote muscle stretching was less in aged rats across all repetitions than in young rats, corroborating the findings of Gajdosik et al. 31. These authors compared the maximal passive force (N) between older and younger women with limited dorsiflexion. They observed that maximal passive force was significantly lower in older women after a stretching program. The authors attributed this outcome to sarcopenia combined with a decrease in calf muscle length related to aging, which would decrease the ability of the calf muscle to withstand passive stretching to the tolerated maximal stretch angle 31.

Willems et al. 32 performed muscle stretching in rats, establishing a 90-degree angle neutral position of the tibio-tarsal joint and maximal dorsiflexion to promote stretching of 40° of the tibio-tarsal joint angle. In the present study, the joint angles were similar to the measures reported by Willems et al. 32. Although the force applied to induce muscle stretching in aged rats was less than that in young rats, there was no intergroup difference in the tibio-tarsal angle joint.

According to Carter et al. 33, during the aging process, the soleus muscle shows greater reductions in biomechanical, morphological and molecular aspects than the gastrocnemius muscle. These authors reported that the soleus of aged rats demonstrated a decline in passive muscle resistive force compared with the soleus of young rats 33. Kodama et al. 15 also observed a decrease in the passive resistive force of the gastrocnemius muscle of aged rats (15 months) submitted to traction tests compared with the gastrocnemius muscle of young rats. These results corroborate the findings presented in the current paper.

Concerning the amount of force necessary to correct the restriction of human ankle joint dorsiflexion, it has been stated that it is necessary to use an average of 9 kg (4.5-13.5 kg) 34. According to the authors of a previous study, the average weight of 40-year-old Japanese men is approximately 65 kg; 9 kg is equivalent to approximately 15% of that weight 11. Using this ratio, the authors reported that 0.3 N is required for eight-week-old female Wistar rats with an average body weight of 200 g to perform ankle dorsiflexion 12. In the present study, the force necessary to stretch the soleus muscle of young female rats was 0.11 N that of aged female rats was 0.08 N. This result may be explained by the fact that the young rats in this study were older (12 weeks) and heavier (311±11 g), requiring less force to stretch the soleus muscle, than those in the previous study.

According to Haus et al. 35, the connective tissue scaffold is an important factor in transferring the force from the contractile units of the muscle to the tendon. However, muscle force transmission and muscle function during aging are altered by glycation-related cross-linking of intramuscular connective tissues 35. Stretching exercises are recognized as important for preventing fibrosis and treat musculoskeletal diseases 2. Thus, it is important to elucidate the effects of stretching exercise in rats by monitoring the force used in the induced muscle elongation and to explain the mechanisms involved in musculoskeletal adaptation related to the aging process.

A limitation of the present study is that fascicle length and pennation angle were not measured, which should be further investigated using ultrasound imaging.

The outcomes of this study show that 1) the device developed was efficient and able to mechanically stretch the soleus muscle of young and aged rats; 2) less force was necessary to stretch the soleus muscle of aged female rats than the soleus muscle of young female rats; and 3) there was no difference between the YG and the AG regarding the tibio-tarsal angle position.

## AUTHOR CONTRIBUTIONS

Zotz TGG, Zotz R, Goossen E and Gomes ARS participated in the device development and experimental design. Guimarães ATB contributed to the statistical analysis. Zotz TGG and Gomes ARS also contributed to the statistical analysis and supervised the entire study. All coauthors read and approved the submitted manuscript.

## Figures and Tables

**Table 1:** Load Cell Calibration

Mass (g)	Load Cell Signal (g)	Record force (N)
0	0	0
9.99	10	0.09±0.00
20.04	20	0.19±0.00
40.07	40	0.39±0.00
90.05	90	0.88±0.00
190.07	190	1.86±0.00
290.14	290	2.84±0.00
475.90	490	4.66±0.00
676.00	690	6.63±0.00
875.90	890	8.59±0.00
1075.90	1090	10.50±0.00

Recorded force data are presented as the mean±SD of seven measurements.

**Table 2:** Force (N) behavior during stretching.

Repetition		Young Group (N)	Aged Group (N)	*p*
1^st^	Initial	0.64±0.05	0.50±0.06	0.99
	Final	0.53±0.05	0.41±0.04	
	Delta	0.11±0.02	0.09±0.03	
2^nd^	Initial	0.65±0.04	0.48±0.05	1.00
	Final	0.55±0.06	0.39±0.05	
	Delta	0.10±0.03	0.09±0.01	
3^rd^	Initial	0.65±0.05	0.51±0.01	1.00
	Final	0.54±0.08	0.41±0.02	
	Delta	0.11±0.04	0.09±0.02	
4^th^	Initial	0.66±0.04	0.46±0.03	0.99
	Final	0.56±0.05	0.38±0.03	
	Delta	0.10±0.04	0.08±0.02	
5^th^	Initial	0.64±0.06	0.49±0.03	0.74
	Final	0.50±0.03	0.41±0.03	
	Delta	0.13±0.03	0.08±0.01	
6^th^	Initial	0.64±0.03	0.48±0.03	0.99
	Final	0.52±0.03	0.39±0.04	
	Delta	0.12±0.04	0.09±0.03	
7^th^	Initial	0.63±0.03	0.48±0.03	0.96
	Final	0.61±0.03	0.40±0.04	
	Delta	0.11±0.03	0.07±0.02	
8^th^	Initial	0.66±0.05	0.46±0.03	0.98
	Final	0.55±0.07	0.39±0.04	
	Delta	0.11±0.05	0.07±0.03	
9^th^	Initial	0.61±0.04	0.47±0.03	1.00
	Final	0.52±0.04	0.38±0.04	
	Delta	0.09±0.02	0.08±0.01	
10^th^	Initial	0.63±0.02	0.48±0.03	0.99
	Final	0.54±0.03	0.40±0.04	
	Delta	0.09±0.02	0.07±0.02	
*p*		*0.00		

Data are presented as the mean±SD (N) of the initial force (N), final force (N), and delta force (final minus initial) measurements recorded during stretching. Delta compared intragroup and intergroup values by repeated measures ANOVA.

* Statistical significance of intergroups (repeated measures ANOVA).
